# Thiamine Level in Type I and Type II Diabetes Mellitus Patients: A Comparative Study Focusing on Hematological and Biochemical Evaluations

**DOI:** 10.7759/cureus.8027

**Published:** 2020-05-08

**Authors:** Adnan Anwar, Muhammad Ahmed Azmi, Jamil Ahmed Siddiqui, Ghazala Panhwar, Farheen Shaikh, Madiha Ariff

**Affiliations:** 1 Stereotactic Radiosurgery/Radiation Oncology, Al-Tibri Medical College, Karachi, PAK; 2 Physiology, Al-Tibri Medical College, Karachi, PAK; 3 Physiology, Al-Tibri Medical College and Hospital, Karachi, PAK; 4 Physiology, Isra University, Karachi, PAK; 5 Biochemistry, Fazaia Ruth Pfau Medical College, Karachi, PAK; 6 Biochemistry, Al-Tibri Medical College, Karachi, PAK; 7 Biochemistry, Al-Tibri Medical College and Hospital, Karachi, PAK; 8 Microbiology, Dr. Ziauddin Hospital, Karachi, PAK; 9 Internal Medicine, Dow University of Health Sciences, Karachi, PAK

**Keywords:** diabetes mellitus type i, diabetes mellitus type ii, thiamine level, vitamin b1.

## Abstract

Objective

Diabetes has been found to be associated with low levels of thiamine stores in the body, as thiamine directly affects carbohydrate metabolism. Amplified renal clearance of thiamine has been found in both type I and type II diabetic patients. It has been shown that high-dose thiamine therapy may have a therapeutic effect on early-stage diabetic nephropathy. The aim of this study was to evaluate various biochemical parameters and serum thiamine levels in type I and type II diabetic patients and compare them with a healthy control group.

Methods

A case-control study was carried out in the diabetic out-patient multi-centers in Karachi. A total of 90 participants were selected by using a non-probability convenient sampling technique and divided into three groups, each with 30 subjects. Group A included healthy non-diabetic subjects, while group B included subjects with type I diabetes mellitus (DM), and group C included subjects with type II DM. After receiving informed consent, blood samples were collected from all the participants for hematological and biochemical evaluation. The duration of the study was eight months.

Results

The study results revealed that the patients with type II DM had significantly higher mean fasting blood sugar (FBS), random blood sugar (RBS), and hemoglobin A1c (HbA1c) levels than those with type I DM or the control group (p<0.001 for all). Furthermore, the patients with type I or II DM had significantly higher mean levels of triglyceride (p<0.001) and total cholesterol (0.013) while significantly lower mean levels of high-density lipoprotein (HDL) (p=0.014) than controls. The study results further revealed that the patients with type I or II DM had significantly lower serum thiamine levels than controls (14.89±4.82 and 7.35±1.90 vs. 69.56±12.75, p<0.001).

Conclusion

The study results revealed that FBS, RBS, HbA1c, triglyceride, and total cholesterol levels were significantly higher in both type I and type II diabetes patients compared to controls. Furthermore, HDL and serum thiamine levels were found to be significantly lower in both type I and type II diabetic patients than in controls.

## Introduction

Thiamine is a water-soluble vitamin that consists of pyrimidine and a thiazole ring with a methylene bridge in between [1]. Thiamine also plays a vital role in the various phases of the intermediary metabolism, as a requisite and an irreplaceable coenzyme. Thiamine functions as an antioxidant and possesses anti-inflammatory properties, and thus affects endothelial function. It may occur in several forms such as free thiamine, thiamine monophosphate (TMP), thiamine diphosphate (TDP), thiamine triphosphate (TTP), and adenosine thiamine triphosphate (ATT). Thiamine plays an equally imperative role in lipid metabolism and is considered to be essential for development in children [2]. The intake of thiamine should be about 1.0-1.4 mg/day for adult men and 0.8-1.1 mg/day for adult women [3]. Thiamine is absorbed chiefly in the duodenum via the thiamine transport protein system. The two transporters, THTR1 and THTR2, are responsible for thiamine transportation and delivery across the body into the tissues [4]. Both THTR1 and THTR2 are found to be in high concentrations in tissues such as the placenta, liver, and kidney; however, THTR1 is also found in skeletal muscles and cardiac tissue [5,6]. Serum and urine thiamine levels are measured via blood and excretion of thiamine metabolites in the urine, respectively [7]. High-performance liquid chromatography (HPLC) is considered to be a simple and precise way of assessing thiamine concentration in blood [8]. Diabetes mellitus (DM) is one of the most critical health issues, and its incidence is increasing rapidly in all age groups. DM is associated with genetic, environmental, and host factors. The two basic processes by which diabetes can occur are autoimmune and metabolic pathways. The prominent risk factors of DM are malabsorption, obesity, inactivity, and hormonal imbalance. DM is associated with atherosclerosis, stroke, renal insufficiency, and insulin shock. Regular exercise and a balanced diet are the prime ways to prevent DM, especially type II DM [9,10]. DM is generally divided into type I (insulin-dependent DM) and type II (insulin-independent DM). Type I DM occurs due to the autoimmune destruction of beta cells of the pancreas and is caused by the failure of pancreatic beta cells to produce insulin, while type II DM, which is more common, results from the development of insulin resistance and decreased insulin receptor sensitivity [9]. Type II DM is more prevalent in developing countries like Pakistan [10]. Diabetes is found to be associated with low levels of thiamine stores in the body, as thiamine directly affects carbohydrate metabolism. Amplified renal clearance of thiamine has been found in both type I and type II diabetic patients [11]. Thiamine deficiency results from long-term use of diuretics, causing a sudden decrease in thiamine excretion that may lead to renal damage [12]. Diabetic nephropathy, which is one of the most serious complications of diabetes, becomes clinically overt with the presence of microalbuminuria, which leads to macroalbuminuria. At this stage, adequate renal replacement therapy is required for kidneys to function efficiently [13]. The presence of microalbuminuria is considered to be indicative of diabetic nephropathy in diabetic patients and also signifies cardiovascular events [14,15]. The progression of diabetic nephropathy in patients with type I DM can be halted by controlling glycemia and hypertension with angiotensinogen-converting enzyme (ACE) inhibitor therapy that reduces microalbuminuria [16]. It has been found that thiamine level is reduced in diabetes as renal clearance of thiamine increases [17]. High-dose thiamine therapy may have a therapeutic effect on early-stage diabetic nephropathy [18]. Thiamine therapy is also suggested to be useful in preventing renal and cardiovascular events in people with type II DM, thereby increasing the quality of life and reducing further complications [19]. This study aimed to evaluate various biochemical parameters and serum thiamine levels in type I and type II diabetic patients and compare them with a healthy control group.

## Materials and methods

Ethical approval was obtained from the authorities at the Al-Tibri Medical College and Hospital for this study The study was conducted for eight months, from January 2019 till August 2019. The study was carried out in the diabetic out-patient multi-centers in Karachi. A total of 90 participants were selected by using a non-probability convenient sampling technique and divided into three groups, each with 30 subjects. Group A was the control group and included healthy non-diabetic subjects, while group B included subjects with type I DM, and group C included subjects with type II DM. All patients with type I and II DM of either gender were included in the study. However, patients using diuretics, those with significant co-morbidities, or those who had undergone major transplant surgeries were excluded from the study. After receiving informed consent from the participants, blood samples were collected from the diabetic clinics of multi-centers in Karachi. The blood samples were collected in heparinized tubes. From each sample, red blood cell (RBC) count, hemoglobin concentration (Hb), hematocrit (Hct), mean corpuscular volume (MCV), mean corpuscular hemoglobin (MCH), mean corpuscular hemoglobin concentration (MCHC), total leucocyte count (TLC), differential leucocyte count (DLC), and platelet count were assessed and analyzed. Blood samples collected in the non-heparinized tube were immediately centrifuged at 2000 rounds per minute (rpm) for 20 minutes. The clear supernatant serum was used for the assessment of various biochemical diagnostic parameters including creatinine, urea, fasting blood sugar (FBS) levels, random blood sugar (RBS) levels, hemoglobin A1c (HbA1c), fasting lipid profile, blood thiamine levels, urinary thiamine levels, and microalbuminuria. The data were entered and analyzed by SPSS Statistics version 20 (IBM, Armonk, NY). Descriptive analysis was reported in terms of mean and standard deviation, whereas inferential analysis was performed by applying a one-way analysis of variance (ANOVA) to compare the means across the three study groups. The significance level was set at a p-value of 0.05.

## Results

The study results revealed differences of statistical significance in the means of all the baseline characteristics except height and temperature between the three study groups: age, body mass index (BMI), weight, heart rate (p<0.001 for all), systolic blood pressure (p=0.001), and diastolic blood pressure (p=0.002); the patients with type I diabetes were younger and had lower BMI and weight than those with type II diabetes or controls, whereas patients with type I and II DM had higher systolic and diastolic blood pressure and heart rate than controls (Table 1).

**Table 1:** Comparison of baseline characteristics of the participants in different groups (n=90) *One-way ANOVA ANOVA: analysis of variance

Characteristics		Mean	Standard deviation	95% confidence interval for mean		P-value*
				Lower bound	Upper bound	
Age, years	Control	41.73	6.88	39.16	44.30	<0.001
	Type I	24.20	6.40	21.81	26.59	
	Type II	42.73	10.52	38.81	46.66	
Body mass index, kg/m2	Control	28.56	7.68	25.69	31.42	<0.001
	Type I	15.63	2.88	14.56	16.70	
	Type II	31.85	5.64	29.75	33.96	
Height, feet and inches	Control	5.18	0.51	4.99	5.37	0.092
	Type I	4.93	0.52	4.73	5.12	
	Type II	5.13	0.38	4.99	5.27	
Weight, kg	Control	66.57	8.11	63.54	69.60	<0.001
	Type I	33.77	6.59	31.31	36.23	
	Type II	74.03	7.68	71.17	76.90	
Systolic blood pressure, mmHg	Control	122.00	10.31	118.15	125.85	0.001
	Type I	129.67	12.99	124.81	134.52	
	Type II	133.67	12.73	128.91	138.42	
Diastolic blood pressure, mmHg	Control	81.00	8.85	77.70	84.30	0.002
	Type I	89.67	13.51	84.62	94.71	
	Type II	91.00	10.94	86.92	95.08	
Heart rate, bpm	Control	71.63	4.97	69.78	73.49	<0.001
	Type I	77.30	8.65	74.07	80.53	
	Type II	78.73	5.57	76.65	80.81	

The male-to-female ratio in the control group was 1:1 (n=15, in both genders); it was 1:2.75 (male: n=8; female: n=22) in type I DM, and 1:2 (male: n=10; female: n=20) in type II DM groups. The study results further revealed differences of statistical significance in FBS and RBS and HbA1c levels between the three study groups (p<0.001 for all); the patients with type II DM had higher FBS, RBS, and HbA1c levels than those with type I DM or controls (Table 2).

**Table 2:** Comparison of serum blood glucose levels and HbA1c (n=90) *One-Way ANOVA HbA1c: hemoglobin A1c; ANOVA: analysis of variance

Serum blood glucose levels		Mean	Standard deviation	95% confidence interval for mean		P-value*
				Lower bound	Upper bound	
Fasting blood glucose, mg/dL	Control	87.10	11.784	82.70	91.50	<0.001
	Type I	151.30	46.032	134.11	168.49	
	Type II	211.77	72.139	184.83	238.70	
Random blood glucose, mg/dL	Control	146.50	28.962	135.69	157.31	<0.001
	Type I	268.33	36.547	254.69	281.98	
	Type II	282.50	45.557	265.49	299.51	
HbA1c, %	Control	5.2067	0.29935	5.0949	5.3184	<0.001
	Type I	7.4933	0.62474	7.2601	7.7266	
	Type II	9.3800	1.97840	8.6413	10.1187	

While comparing the lipid profile across the three study groups, it was seen that the mean levels of triglycerides (p<0.001), high-density lipoprotein (HDL, p=0.014), and total cholesterol (p=0.013) showed differences of statistical significance; the patients with type I or II DM had higher levels of triglyceride and total cholesterol and lower levels of HDL than controls (Table 3).

**Table 3:** Comparison of lipid profile between groups (n=90) *One-Way ANOVA ANOVA: analysis of variance

Lipid profile		Mean	Standard deviation	95% confidence interval for mean		P-value*
				Lower bound	Upper bound	
Triglycerides, mg/dL	Control	117.43	18.765	110.43	124.44	<0.001
	Type I	169.43	57.252	148.06	190.81	
	Type II	152.17	56.966	130.90	173.44	
Low-density lipoprotein, mg/dL	Control	110.05	22.135	101.78	118.32	0.237
	Type I	118.13	16.113	112.12	124.15	
	Type II	113.63	16.081	107.63	119.64	
High-density lipoprotein, mg/dL	Control	45.63	7.374	42.88	48.39	0.014
	Type I	40.40	6.709	37.89	42.91	
	Type II	41.87	6.852	39.31	44.43	
Total cholesterol, mg/dL	Control	177.83	16.140	171.81	183.86	0.013
	Type I	202.20	34.191	189.43	214.97	
	Type II	189.27	38.602	174.85	203.68	

**Figure 1:**
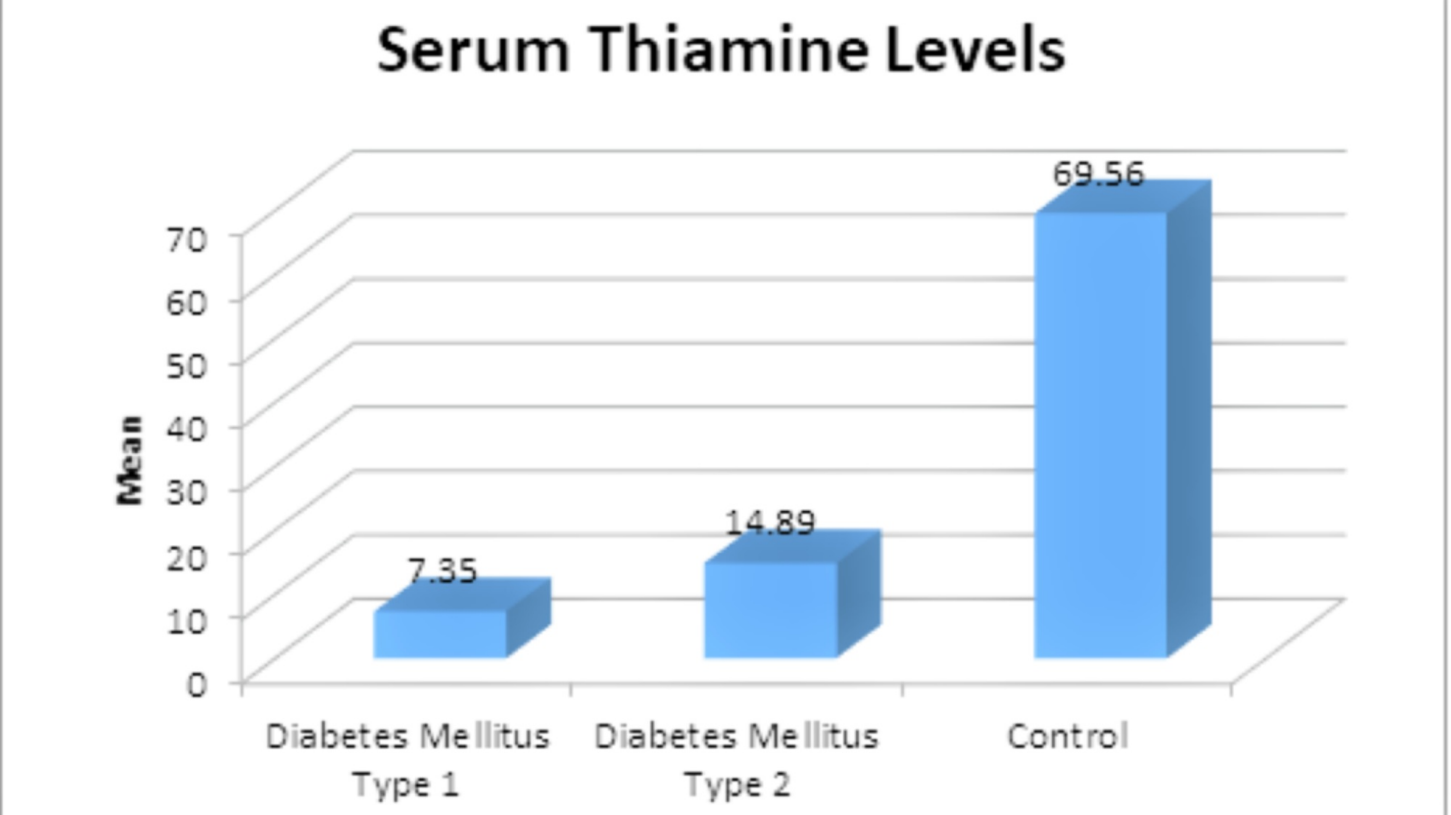
Serum thiamine levels among participants in different groups

The study results further revealed that the patients with type I or II DM had statistically significantly lower serum thiamine levels than controls (14.89±4.82 and 7.35±1.90 vs. 69.56±12.75, p<0.001) (Figure 1).

## Discussion

We found significantly higher levels of RBS and FBS and HbA1c in subjects with type I or type II DM, as compared to the subjects in the control group. Also, increased levels of triglycerides and total cholesterol were found in participants with type I and type II DM as compared to subjects in control groups. Additionally, the study results revealed significantly lower mean HDL levels in patients with type I and type II DM as compared to the subjects in the control group. A noteworthy finding of our study was that the mean serum thiamine levels were significantly lower in patients with type I and type II DM than in controls. As expected, our results showed that both FBS and RBS levels were significantly higher in both type I and II DM patients than in controls, a finding well in line with published literature. An earlier study conducted in 2015 reported glucose levels to be significantly higher in patients with type 1 DM than in controls (p=0.001) [20]. Similarly, and as expected, the study findings further revealed that HbA1c was significantly higher in both type I and type II DM patients than in controls. An earlier study carried out in 2003 also reported patients with DM having higher HbA1c levels than subjects without DM (p=0.002). HbA1c has also been suggested to be a highly specific and convenient screening and diagnostic tool for diabetes [21]. The study results also showed that triglycerides and cholesterol were significantly higher in both types I and II DM patients than in controls. Triglycerides and cholesterol have been previously reported to be significantly higher in patients with type I DM than in controls (p=0.008) [19]. Likewise, triglycerides have also been reported to be significantly higher in patients with type II DM than in controls (p<0.001) [22]. The study results further revealed that HDL level was found to be significantly lower in patients with type I and type II DM compared to the control group. Similarly, an earlier study conducted in 2016 also found HDL levels to be significantly decreased in patients with type I DM than in controls (p=0.005) [19]. Likewise, another study done in 2013 reported HDL levels to be significantly decreased in type II diabetic patients than in controls (p<0.001) [22]. The study results also demonstrated microalbuminuria values to be higher in both type I and type II DM patients than in controls. Microalbuminuria level has previously been found to be significantly higher in patients with DM type I than in controls (p=0.02) [19]. These findings highlight the importance of reducing microalbuminuria in such patients to prevent any long-term complications of deranged protein excretion in diabetes, as literature has also reported a 50% reduction in microalbuminuria in type II DM patients to significantly lower the risk of cardiovascular events [adjusted Odds Ratio (AOR): 0.41, 95% CI: 0.15- 0.96] [23]. Interestingly, our findings further revealed that serum thiamine levels were significantly lower in both type I and type II DM patients than in controls. An earlier study done in 2016 also reported thiamine levels to be significantly lower in patients with type I DM than in controls (p=0.002) [19]. Another study carried out in 2007 also found plasma thiamine concentration to be significantly lower in type I and type II diabetic patients (p<0.001 for both) than in normal controls [24]. Similarly, another study conducted in 2012 reported blood thiamine concentration to be significantly decreased in patients with DM type I and II than in controls (p<0.001) [11]. Likewise, plasma thiamine chloride and thiamine monophosphate levels have been reported to be significantly lower in patients with type II DM than in controls (p<0.001) [22]. Notably, contrary to these findings, a case-control study carried out in 2015 reported plasma thiamine concentration to be significantly higher in type II DM patients, both with and without microalbuminuria, than in controls (p<0.0001) [16]. A role of functional thiamine deficiency in the development of hyperglycemia-related pathology has also been documented earlier [25]. Renal clearance of thiamine has also been reported to increase by 24-fold in type I diabetic patients and by 16-fold in type II diabetic patients, inevitably culminating in thiamine deficiency, which implicates the need for thiamine replacement therapy in such patients [24]. Such supplementation has been reported to result in beneficial outcomes in patients suffering from DM. An earlier study conducted in 2012 reported high-dose thiamine therapy in patients with type II DM resulted in significantly reduced microalbuminuria and glycated hemoglobin [26]. Another study conducted in 2011 reported thiamine administration in patients with type II DM resulted in significantly decreased glucose levels (p=0.024) [27]. Thiamine supplementation has also been reported to prevent the reappearance of diabetic complications for over six years in a continuous open trial [17]. A review article published in 2011 reported that thiamine administration prevents the formation of harmful by-products of glucose metabolism, reduces oxidative stress, and improves endothelial function [28]. Another review article published in 2011 reported that thiamine supplementation reverses increased urinary albumin excretion in patients with type II DM and microalbuminuria [29]. Our study demonstrated that patients with type I and II DM had lower levels of serum thiamine. Nevertheless, this study can have selection bias due to a non-probability sampling technique and observer bias. Hence, prospective studies with a probability sampling technique are recommended to elaborate on this association in larger samples to get more precise outcomes.

## Conclusions

The study results revealed that FBS, RBS, HbA1c, triglyceride, and total cholesterol levels were significantly higher in both type I and type II diabetic patients compared to controls. Furthermore, HDL and serum thiamine levels were found to be significantly lower in both type I and type II diabetic patients than in controls. Being an observational study, the study findings, although suggestive of associations of blood glucose levels and HbA1c with blood thiamine level, could not establish a temporal association between the aforementioned variables. For this purpose, a cohort study design is warranted.
